# Supplementary material for: Selected miRNA and Psoriasis—Cardiovascular Disease (CVD)—Overweight/Obesity Network—A Pilot Study
Source: Int J Mol Sci. 2023 Sep 10;24(18):13916. doi: 10.3390/ijms241813916 (PMC10530775; doi:10.3390/ijms241813916)
Supplement: Supplementary file 1 [file ijms-24-13916-s001.zip › ijms-2568522-supplementary.pdf]

Table S1. miRNA-disease network.

Disease	Degree	Betweenness	miR-22-3p	miR-133a-3p	miR-146a-5p	miR-369-3p	Let-7b-5p
Carcinoma, Hepatocellular	5	421.5268	+	+	+	+	+
Colorectal Carcinoma	5	421.5268	+	+	+	+	+
Leukemia, Myeloid, Acute	4	171.2146	+	-	+	+	+
Breast cancer	4	171.2146	+	-	+	+	+
Cardiovascular Diseases [unspecific]	4	375.0937	+	+	+	-	+
Muscular disorder	4	171.2146	+	-	+	+	+
Pancreatic cancer	4	171.2146	+	-	+	+	+
Prostate cancer	4	171.2146	+	-	+	+	+
Lung Neoplasms	4	375.0937	+	+	+	_	+
Pancreatic Neoplasms	4	375.0937	+	+	+	-	+
Carcinoma, Renal Cell	4	375.0937	+	+	+	-	+
Prostate Neoplasms	4	375.0937	+	+	+	-	+
Myocardial Infarction	4	375.0937	+	+	+	-	+
Ovarian Neoplasms	4	375.0937	+	+	+	-	+
Gastric Neoplasms	4	375.0937	+	+	+	-	+
Heart Failure	4	375.0937	+	+	+	-	+
Carcinoma, Lung, Non-Small-Cell	4	375.0937	+	+	+	-	+
Breast Neoplasms	4	375.0937	+	+	+	-	+
Atherosclerosis	4	375.0937	+	+	+	-	+
Hypertension	4	375.0937	+	+	+	-	+
Asthma	4	375.0937	+	+	+	-	+
Colon Neoplasms	4	375.0937	+	+	+	-	+
Inflammation	4	375.0937	+	+	+	-	+
Leukemia, Lymphoblastic, Acute	3	139.3231	+	+	-	-	+
Cardiomyopathy, Dilated	3	131.8566	+	+	-	-	+
Colorectal cancer	3	139.3231	+	-	+	-	+
Hematological disease	3	139.3231	+	-	+	-	+
hepatocellular carcinoma	3	139.3231	+	-	+	-	+
Leukemia, chronic lymphatic	3	139.3231	+	-	+	-	+
Lung cancer	3	139.3231	+	-	+	-	+
Medulloblastoma	3	139.3231	+	+	-	-	+
Melanoma and neural system tumor syndrome	3	38.37491	+	-	+	+	+
Multiple Myeloma	3	139.3231	+	-	+	-	+
Ovarian cancer	3	139.3231	+	-	+	-	+
Carcinoma, Thyroid, Papillary	3	91.2148	-	-	+	+	+
Squamous Cell Carcinoma, Head and Neck	3	139.3231	+	-	+	-	+
Stroke	3	139.3231	+	-	+	-	+
Alzheimer Disease	3	139.3231	+	-	+	-	+
Muscular Dystrophy, Duchenne	3	55.65512	+	-	+	+	-
Leukemia, Myeloid, Chronic	3	73.51632	+	-	+	+	-
Psoriasis	3	73.51632	+	-	+	+	-
Squamous Cell Carcinoma, Oral	3	215.7641	+	+	+	-	-
Vascular Disease [unspecific]	3	139.3231	+	-	+	-	+
Sepsis	3	215.7641	+	+	+	-	-
Diabetes Mellitus, Type 2	3	215.7641	+	+	+	-	-
Preeclampsia	3	139.3231	+	-	+	-	+
Ischemia-Reperfusion Injury	3	263.2435	-	+	+	-	+
Coronary Artery Disease	3	215.7641	+	+	+	-	-
Amyotrophic Lateral Sclerosis	3	139.3231	+	-	+	-	+
Graves Disease	3	139.3231	+	-	+	-	+
Pulmonary Hypertension	3	139.3231	+	-	+	-	+
Thyroid Neoplasms	3	139.3231	+	-	+	-	+
Melanoma	3	139.3231	+	-	+	-	+
Multiple Sclerosis	3	73.51632	+	-	+	+	-

**Myelodysplastic Syndromes**	3	139.3231	+	-	+	-	+
**Liver Neoplasms**	3	139.3231	+	-	+	-	+
**Carcinoma, Colon**	3	263.2435	-	+	+	-	+
**Esophageal Neoplasms**	3	139.3231	+	+	+	-	-
**Glioblastoma**	3	139.3231	+	-	+	-	+
**Neoplasms [unspecific]**	3	139.3231	+	-	+	-	+
**Head And Neck Neoplasms**	3	263.2435	-	+	+	-	+
**Carcinoma, Cervical**	3	215.7641	+	+	+	-	-
**Carcinoma, Endometrial**	3	55.65512	+	+	-	+	-
**Carcinoma, Prostate**	3	139.3231	+	-	+	-	+
**Carcinoma, Adrenocortical**	2	63.95487	-	-	+	-	+
**Lymphoma, Burkitt**	2	63.95487	-	-	+	-	+
**Cancer**	2	63.95487	-	-	+	-	+
**Cardiac hypertrophy**	2	63.95487	-	-	+	-	+
**Cervical cancer, somatic**	2	63.95487	-	-	+	-	+
**Muscular Dystrophy, Facioscapulohumeral**	2	69.81319	-	+	-	-	+
**Miyoshi myopathy**	2	63.95487	-	-	+	-	+
**Muscular dystrophy, limb-girdle, type 2A**	2	63.95487	-	-	+	-	+
**Myopathy, nemaline, 3**	2	8.18189	-	-	-	+	+
**Neuroblastoma**	2	63.95487	-	-	+	-	+
**Neurodegeneration**	2	63.95487	-	-	+	-	+
**Parkinson Disease**	2	25.56149	+	-	-	-	+
**Uveal Melanoma**	2	63.95487	-	-	+	-	+
**Intrahepatic Cholangiocarcinoma**	2	49.80675	+	-	+	-	-
**Leukemia/lymphoma, chronic B-cell**	2	49.80675	+	-	+	-	-
**Melanoma, cutaneous malignant, 2**	2	49.80675	+	-	+	-	-
**Rhabdomyosarcoma**	2	36.48195	+	+	-	-	-
**Adenocarcinoma, Pancreatic Ductal**	2	129.4754	-	+	+	-	-
**Rheumatoid Arthritis**	2	49.80675	+	-	+	-	-
**Leukemia, Lymphocytic, Chronic, B-Cell**	2	49.80675	+	-	+	-	-
**Leukemia-Lymphoma, Precursor T-Cell Lymphoblastic**	2	63.95487	-	-	+	-	+
**Endometriosis**	2	25.56149	+	-	-	-	+
**Diabetes Mellitus**	2	129.4754	-	+	+	-	-
**Crohn Disease**	2	63.95487	-	-	+	-	+
**Stroke, Ischemic**	2	63.95487	-	-	+	-	+
**Squamous Cell Carcinoma, Esophageal**	2	129.4754	-	+	+	-	-
**Systemic Lupus Erythematosus**	2	49.80675	+	-	+	-	-
**Muscle Atrophy**	2	49.80675	+	-	+	-	-
**Leukemia**	2	63.95487	-	-	+	-	+
**Hepatitis C Virus Infection**	2	63.95487	-	-	+	-	+
**Aortic Stenosis**	2	36.48195	+	+	-	-	-
**Hepatitis B Virus Infection**	2	49.80675	+	-	+	-	-
**Carcinoma, Urothelial, Upper Tract**	2	25.56149	+	-	-	-	+
**Epilepsy**	2	63.95487	-	-	+	-	+
**Chronic Kidney Disease**	2	129.4754	-	+	+	-	-
**Hirschsprung Disease**	2	19.07804	-	-	+	+	-
**Carcinoma, Pancreatic**	2	36.48195	+	+	-	-	-
**Ischemic Heart Disease**	2	129.4754	-	+	+	-	-
**Acute Coronary Syndrome**	2	129.4754	-	+	+	-	-
**Periodontitis**	2	63.95487	-	-	+	-	+
**Obesity**	2	63.95487	-	-	+	-	+
**Neurodegenerative Diseases [unspecific]**	2	63.95487	-	-	+	-	+
**Cholangiocarcinoma**	2	49.80675	+	-	+	-	-
**Nasopharyngeal Neoplasms**	2	63.95487	-	-	+	-	+
**Digestive System Neoplasms**	2	63.95487	-	-	+	-	+

**Lymphoma, B-Cell**	2	49.80675	+	-	+	-	-
**Endometrial Neoplasms**	2	63.95487	-	-	+	-	+
**Intracranial Aneurysm**	2	129.4754	-	+	+	-	-
**Kidney Diseases [unspecific]**	2	129.4754	-	+	+	-	-
**Carotid Atherosclerosis**	2	129.4754	-	+	+	-	-
**Carcinoma, Oral**	2	63.95487	-	-	+	-	+
**Carcinoma, Ovarian**	2	49.80675	+	-	+	-	-
**Kaposi Sarcoma**	2	63.95487	-	-	+	-	+
**Macular Degeneration**	2	63.95487	-	-	+	-	+
**Bladder Neoplasms**	2	129.4754	-	+	+	-	-
**Carcinoma, Breast**	2	129.4754	-	+	+	-	-
**Hypertrophy**	2	69.81319	-	+	-	-	+
**Myocardial Ischemic-Reperfusion Injury**	2	63.95487	-	-	+	-	+
**Glioma**	2	69.81319	-	+	-	-	+
**Liver Cirrhosis**	2	49.80675	+	-	+	-	-
**Lymphoma**	2	49.80675	+	-	+	-	-
**Arteriosclerosis Obliterans**	2	36.48195	+	+	-	-	-
**Prion Diseases**	2	63.95487	-	-	+	-	+
**Influenza**	2	63.95487	-	-	+	-	+
**Chronic Inflammation**	2	63.95487	-	-	+	-	+
**Allergy**	2	49.80675	+	-	+	-	-
**Adenocarcinoma, Lung**	2	63.95487	-	-	+	-	+
**Carcinoma, Gastric**	2	36.48195	+	+	-	-	-
**Inflammatory Bowel Diseases**	2	129.4754	-	+	+	-	-
**Atrial Fibrillation**	2	129.4754	-	+	+	-	-
**Cardiac Myocyte Injury**	2	129.4754	-	+	+	-	-
**Huntington Disease**	2	49.80675	+	-	+	-	-
**Osteosarcoma**	2	36.48195	+	+	-	-	-
**Astrocytoma**	2	49.80675	+	-	+	-	-
**Carcinoma, Renal Cell, Clear-Cell**	2	63.95487	-	-	+	-	+
